# On Spencer’s displacement function approach for problems in second-order elasticity theory

**DOI:** 10.1177/10812865221096771

**Published:** 2022-06-10

**Authors:** APS Selvadurai

**Affiliations:** Department of Civil Engineering and Applied Mechanics, McGill University, Montreal, QC, Canada

**Keywords:** Second-order elasticity, Spencer’s displacement function, rubber-like elastic materials, Kelvin’s internal force problem, Love’s doublet problem, Boussinesq’s problem

## Abstract

The paper describes the displacement function approach first proposed by AJM Spencer for the formulation and solution of problems in second-order elasticity theory. The displacement function approach for the second-order problem results in a single inhomogeneous partial differential equation of the form $E^{4}\psi (x)=f(x)$, where $E^{2}$ is Stokes’ operator and f(x) depends only on the first-order or the classical elasticity solution. The second-order isotropic stress p(x) is governed by an inhomogeneous partial differential equation of the form $\nabla^{2}p(x)=g(x)$, where $\nabla^{2}$ is Laplace’s operator and g(x) depends only on the first-order or classical elasticity solution. The introduction of the displacement function enables the evaluation of the second-order displacement field purely through its derivatives and avoids the introduction of arbitrary rigid body terms normally associated with formulations where the strains need to be integrated. In principle, the displacement function approach can be systematically applied to examine higher-order effects, but such formulations entail considerable algebraic manipulations, which can be facilitated through the use of computer-aided symbolic mathematical operations. The paper describes the advances that have been made in the application of Spencer’s fundamental contribution and applies it to the solution of Kelvin’s concentrated force, Love’s doublet, and Boussinesq’s problems in second-order elasticity theory.

## 1. Introduction

Rubber-like elastic materials are characterized by their ability to sustain large strains without fracture and damage. The seminal works of RS Rivlin and co-workers presented a comprehensive and systematic approach to the description of hyperelastic materials, commencing with the formulation of constitutive relations by appeal to the theory of invariants, the solution of benchmark problems involving homogeneous strains, inflation and eversion of annular regions, torsion and bending of prismatic bodies, and performing experiments to validate the mathematical approach. References to these accomplishments are also contained in the collected works of RS Rivlin edited by Barenblatt and Joseph [1] and well documented [2–20] in terms of the contribution of these works to the overall advancement of non-linear continuum mechanics and, in particular, the development of constitutive equations for rubber-like elastic materials [21–35].

When dealing with hyperelastic materials, it is essential to consider the mathematical formulations and the relevant strain energy functions that can accommodate large strains in a consistent fashion. In addition, the realm of application of the hyperelasticity theory is also important. For example, the form of a strain energy function needed to model very large strains in biological tissues [36–43], and may have little relevance to applications dealing with the mechanics of industrial rubber mountings that experience moderately large strains and vice versa [44–51]. A case in point relates to the experiments reported by Rivlin [52] on the torsion of a pure gum rubber cylinder measuring 53.85 mm in diameter and 25.4 mm in length. With these dimensions, the problem is that of the torsion of a disk made of gum rubber and the torque required to induce measurable large strains can be substantial, to the extent that tensile tearing or even surface instabilities [53] can materialize.

The equations governing large strain behavior of rubber-like materials are highly non-linear and unless recourse is made to computational approaches [54–59], the possibility of obtaining analytical solutions to boundary value problems involving the complete non-linear hyperelasticity formulation with generalized forms of the strain energy function is perhaps unrealistic. For this reason, the early research applications in the area of finite elasticity also focused on developing solutions to problems through the method of successive approximations, where the first-order solution corresponded to the relevant classical elasticity solution. The mathematical developments in this area commence with the studies by Signorini [60–62], Mişicu [63], Stoppelli [64], Rivlin [65], Sheng [66], and Grioli [67]. The notion of higher-order theories of elasticity that can be obtained by the suitable expansion of the strains in terms of a small parameter is also discussed by Murnaghan [68,69] but without the development of a formal approach for the solution of boundary value problems. Also, it should be noted that the second-order effects of interest to this paper result from the manifestations of large strains in the hyperelastic medium as opposed to non-linearities that result from purely large deflections and large rotations of a medium that maintains small strain elastic behavior (e.g., *the elastica*). An appraisal of the work of Signorini is also provided by Capriz and Podio-Guidugli [70]. Reviews of the topic of second-order elasticity are given in Truesdell and Noll [7], Green and Adkins [8], Spencer [9] and Rivlin [65], and extensive developments and applications of the theory of second-order elasticity to boundary value problems are documented in the literature cited. For example, Adkins et al. [71] presented a complete development of the plane strain problem in finite elasticity in generalized curvilinear coordinates and with no restriction on the form of the strain energy function. Complex variable techniques [72,73] were used to solve certain benchmark plane strain problems. Adkins et al. [71] also discuss the application of the method of successive approximations, where the first-order approximation relates to the plane strain problem in classical elasticity and the second-order elasticity ensues. Green and Shield [74] continued the developments in the Adkins et al. [71] generalized tensor formulation of the second-order problem applicable to arbitrary forms of the strain energy function. A complex potential function approach was then used to arrive at the relevant integral equations that can be used to examine problems of axial tension and torsion in prismatic bodies that included elliptical cylinders. Adkins and Green [75] presented a complex variable approach to the study of two-dimensional problems in second-order elasticity theory. Blackburn and Green [76] also used two complex potential functions for the analysis of the second-order torsion problem and extended the studies to include bending of a cylinder. Blackburn [77] subsequently extended the work to include the torsion of a region bounded by a single closed curve of a transversely isotropic incompressible elastic material. The second-order bending of an isotropic incompressible cylinder by terminal couples was also examined by Blackburn [78] who reduced the problem to the solution of a single boundary value for two complex potential functions and the first-order problem corresponded to the classical boundary value problem. The axial extension that occurs during the twisting of cylinder was observed by Rivlin [52]. The problem was extended by Green [79] to include arbitrary forms of the strain energy function. The torsion problem was also examined by Sheng [66] using a stress function technique. Green and Spratt [80] investigated the second-order effects in the deformation of both compressible and incompressible elastic bodies and considered the torsion of a solid of revolution. A stress function is used to satisfy the incompressibility condition and equations of equilibrium for second-order elasticity in the axial and radial directions give rise to an inhomogeneous bi-harmonic equation for the stress function. Specific results for the torsion of a cone were presented in exact closed form and the second-order normal force generated during first-order torsion was evaluated explicitly. Application of complex variable methods to the study of two-dimensional problems in finite elasticity is discussed by Adkins et al. [81] and the use of the procedures to the formulation of problems in second-order elasticity for both compressible and incompressible elastic solids is also briefly discussed. The work of Carlson and Shield [82] continued the application of the successive approximation technique to include third-order effects applicable to a general class of plane problems of hyperelastic solids and specific solutions to boundary value problems are given for materials with a strain energy function of the Mooney–Rivlin type. Extensive use of complex variable methods is employed to develop the higher-order solutions to a range of problems of engineering interest. Chan and Carlson [83] examined the second-order incompressible elastic torsion and used, through several changes of the dependent variables, a direct procedure that reduces the second-order torsion problem to the solution of a two-dimensional classical linear elasticity problem without a *pseudo body force* term. The procedure was used to examine the second-order torsion of a bar with a square cross-section. Hill [84] re-examined the approach proposed by Chan and Carlson [83] and proved that the technique is completely general if the strain energy function for the incompressible elastic material is a symmetric function of the remaining principal invariants.

The application of a displacement function approach for the analysis of the second-order problem for incompressible elastic materials was first identified by Spencer in 1968 (see Appendix 1). A formal development of the application of the displacement function technique to axisymmetric problems in second-order elasticity for incompressible elastic materials was presented by Selvadurai and Spencer [85] and a collection of applications to both axisymmetric and two-dimensional problems in incompressible elasticity were presented by Selvadurai [86]. The strain energy function that was adopted for the analysis of problems in second-order elasticity was of the Mooney–Rivlin form and considering the expansion of the response functions in the general constitutive relationship for isotropic incompressible elastic materials, it can be shown that the Mooney–Rivlin form of the strain energy function is sufficient for describing the second-order effects in incompressible elastic materials. The basic methodology proposed by Spencer was adopted to formulate plane strain problems in second-order elasticity theory [87,88], the torsion of annular regions [89], and the axisymmetric loading of a rigid spherical inclusion in an elastic solid of infinite extent [90]. The studies were extended [91] to include axisymmetric problems dealing with spherical cavities and rigid spherical inclusions embedded in incompressible elastic media.

Other approaches have been used for the formulation and solution of problems in second-order elasticity theory. For example, Shield [92] used an energy method with second-order effects and applied it to examine the response of bars composed of compressible hyperelastic materials and subject to the action of torsion under an initial tension. Choi and Shield [93] used an inverse deformation approach to examine the category of second-order elasticity problems involving both the indentation of half-space regions and problems related to an infinite elastic solid containing a spherical cavity. Carroll and Rooney [94] utilize procedures whereby the solution of the second-order elasticity problem can be further facilitated by adopting suitable representations of the second-order incompressibility condition. Carroll and Rooney [95] also used the Strain Potential approach proposed by Love [96] to develop the second-order solution to Lord Kelvin’s problem [97] (see also [98–105]), which was originally developed and formulated in terms of Spencer’s displacement function by Selvadurai [86]. The second-order problem of the torsional indentation of an incompressible elastic half-space by a bonded flat circular punch was considered in the paper by Lindsay [106]. Goodman and Naghdi [107] adopt displacement functions that are similar to the Neuber–Papkovich representations for the solution of problems in linear elasticity [98–105,108] to examine the use of displacement functions in second-order elasticity theory for compressible elastic materials, and present solutions to certain plane strain problems in second-order elasticity theory involving compressible elastic materials. Lindsay [109] also considered the problem of the torsion of an incompressible slab; the problem is formulated by recourse to Weber–Orr transforms that are numerically inverted to produce relevant numerical results. Guo and Kaloni [110] have presented exact closed form solutions to the second-order elasticity problem for a compressible elastic half-space, which is subjected to a non-uniform shear load. The approach used involves the direct application of integral transform techniques to solve the analogous first-order or classical elasticity problem. In addition to the application of the theory of second-order elasticity to the study of three-dimensional problems, the method of successive approximations was applied by Kydoniefs and Spencer [111] to examine the finite inflation of a toroidal membrane. Similar successive approximation techniques were used by Kydoniefs [112] to examine elastic membrane problems. The scope of second-order elasticity in terms of potential applications to rubber-like materials in a technological setting makes the development of analytical approaches a worthwhile exercise.

In this study, we illustrate the application of Spencer’s displacement function approach for the formulation and solution of the second-order problem related to Kelvin’s fundamental problem, which deals with the application of a concentrated force at the interior of an incompressible elastic medium of infinite extent. The strain energy function for the elastic medium is assumed to be of the Mooney–Rivlin form. In the case of an incompressible elastic medium, the similarity between Kelvin’s problem for the concentrated force acting at the interior of an infinite space region and Boussinesq’s problem for a normal force acting at the surface of an elastic half-space is well known [99–101,103,113,114]. The displacement function approach gives explicit solutions to Kelvin’s problem and the methodology is also applied to develop a formal second-order solution to Love’s doublet problem and Boussinesq’s problem for a concentrated normal force on the surface of a half-space.

## 2. Governing equations

We follow the developments documented in Green and Adkins [8], Green and Spratt [80], Selvadurai and Spencer [85], and Selvadurai [86] and adopt the presentation for *axially symmetric problems* in terms of the displacement function approach. We denote particles in the reference configuration by (R,Θ,Z) and particles in the deformed configuration by (r,θ,z). The matrix of deformation gradients in the directions of R,Θ,Z is given by:

(1) $$F=\left(\begin{array}{ccc} \frac{\partial r}{\partial R} & 0 & \frac{\partial r}{\partial Z}\\ 0 & \frac{r}{R} & 0\\ \frac{\partial z}{\partial R} & 0 & \frac{\partial z}{\partial Z} \end{array}\right).$$

We consider incompressible elastic materials for which,

(2) $$\det\;F=\frac{r}{R}\left(\frac{\partial r}{\partial R}\frac{\partial z}{\partial Z}-\frac{\partial r}{\partial Z}\frac{\partial z}{\partial R}\right)=1.$$

The matrix of physical components of the left Cauchy–Green strain tensor, referred to the (r,θ,z) coordinates, is:

(3) $$B=FF^{T}=\left(\begin{array}{ccc} {\left(\frac{\partial r}{\partial R}\right)}^{2}+{\left(\frac{\partial r}{\partial Z}\right)}^{2} & 0 & \frac{\partial r}{\partial R}\frac{\partial z}{\partial R}+\frac{\partial r}{\partial Z}\frac{\partial z}{\partial Z}\\ 0 & \frac{r^{2}}{R^{2}} & 0\\ \frac{\partial r}{\partial R}\frac{\partial z}{\partial R}+\frac{\partial r}{\partial Z}\frac{\partial z}{\partial Z} & 0 & {\left(\frac{\partial z}{\partial R}\right)}^{2}+{\left(\frac{\partial z}{\partial Z}\right)}^{2} \end{array}\right).$$

and, using equation (2), the inverse of equation (3) can be written as:

(4) $$B^{-1}=\left(\begin{array}{ccc} \frac{r^{2}}{R^{2}}\left[{\left(\frac{\partial z}{\partial R}\right)}^{2}+{\left(\frac{\partial z}{\partial Z}\right)}^{2}\right] & 0 & -\frac{r^{2}}{R^{2}}\left[\frac{\partial r}{\partial R}\frac{\partial z}{\partial R}+\frac{\partial r}{\partial Z}\frac{\partial z}{\partial Z}\right]\\ 0 & {\left[\frac{\partial r}{\partial R}\frac{\partial z}{\partial Z}-\frac{\partial r}{\partial Z}\frac{\partial z}{\partial R}\right]}^{2} & 0\\ -\frac{r^{2}}{R^{2}}\left[\frac{\partial r}{\partial R}\frac{\partial z}{\partial R}+\frac{\partial r}{\partial Z}\frac{\partial z}{\partial Z}\right] & 0 & \frac{r^{2}}{R^{2}}\left[{\left(\frac{\partial r}{\partial R}\right)}^{2}+{\left(\frac{\partial r}{\partial Z}\right)}^{2}\right] \end{array}\right).$$

The basis for the method of successive approximations is that the displacement field can be expanded in a power series in terms of a small parameter, ε, which would naturally evolve in the analysis of the first-order problem; we assume that for axisymmetric states of deformation, the coordinates (r,θ,z) can be represented in the form:

(5) $$\begin{array}{cc} r= & R+\varepsilon u_{1}\left(R,Z\right)+\varepsilon^{2}u_{2}\left(R,Z\right)+O\left(\varepsilon^{3}\right),\\ \theta = & \Theta ,\\ z= & Z+\varepsilon w_{1}\left(R,Z\right)+\varepsilon^{2}w_{2}\left(R,Z\right)+O\left(\varepsilon^{3}\right), \end{array}$$

where the suffixes 1 and 2 refer to the first-order and second-order components, respectively. Using equation (5) in equation (2), we obtain the first- and second-order incompressibility conditions as follows:

(6) $$\begin{array}{c} \frac{\partial u_{1}}{\partial R}+\frac{u_{1}}{R}+\frac{\partial w_{1}}{\partial Z}=0,\\ \frac{\partial u_{2}}{\partial R}+\frac{u_{2}}{R}+\frac{\partial w_{2}}{\partial Z}=\frac{u_{1}^{2}}{R^{2}}+\frac{\partial u_{1}}{\partial Z}\frac{\partial w_{1}}{\partial R}-\frac{\partial u_{1}}{\partial R}\frac{\partial w_{1}}{\partial Z}=G\left(R,Z\right). \end{array}$$

For an isotropic incompressible elastic solid, the general form of the constitutive equation for the symmetric contravariant stress tensor T [80] can be reduced to the form:

(7) $$T=-pI+\Phi_{1}B+\Phi_{-1}B^{-1},$$

where $\Phi_{i}(I_{1},I_{2})(i=1,-1)$ are the scalar functions of the principal invariants $I_{1}$ and $I_{2}$ of the strain tensor B, p is a scalar pressure and, for incompressibility, $I_{3}=1$. We assume that the isotropic stress and the stress tensor can also be expanded in power series in terms of the parameter ε in the forms:

(8) $$p=\sum_{n=1}^{\infty }\varepsilon^{n}p_{n}\left(R,Z\right);\;T=\sum_{n=1}^{\infty }\varepsilon^{n}T_{n}\left(R,Z\right).$$

The first-order constitutive equation can be reduced to the forms:

(9) $$\begin{array}{cc} T_{\mathrm{rr}}^{\left(1\right)}= & -p_{1}+2\mu \frac{\partial u_{1}}{\partial R},\\ T_{\theta \theta }^{\left(1\right)}= & -p_{1}+2\mu \frac{u_{1}}{R},\\ T_{\mathrm{zz}}^{\left(1\right)}= & -p_{1}+2\mu \frac{\partial w_{1}}{\partial Z},\\ T_{\mathrm{rz}}^{\left(1\right)}= & \mu \left(\frac{\partial u_{1}}{\partial Z}+\frac{\partial w_{1}}{\partial R}\right), \end{array}$$

where $\mu (=2(C_{1}+C_{2}))$ is the linear elastic shear modulus, and the constants $C_{1}$ and $C_{2}$ can be identified with the material constants characterizing the Mooney–Rivlin form of the strain energy function:

(10) $$W\left(I_{1},I_{2}\right)=C_{1}\left(I_{1}-3\right)+C_{2}\left(I_{2}-3\right).$$

The constitutive equations for the second-order stress components can be written as:

(11) $$\begin{array}{cc} T_{\mathrm{rr}}^{\left(2\right)}= & -p_{2}+2\mu \frac{\partial u_{2}}{\partial R}+t_{\mathrm{rr}},\\ T_{\theta \theta }^{\left(2\right)}= & -p_{2}+2\mu \frac{u_{2}}{R}+t_{\theta \theta },\\ T_{\mathrm{zz}}^{\left(2\right)}= & -p_{2}+2\mu \frac{\partial w_{2}}{\partial Z}+t_{\mathrm{zz}},\\ T_{\mathrm{rz}}^{\left(2\right)}= & \mu \left(\frac{\partial u_{2}}{\partial Z}+\frac{\partial w_{2}}{\partial R}\right)+t_{\mathrm{rz}}, \end{array}$$

and the components $t_{\mathrm{rr}}\;,t_{\theta \theta },t_{\mathrm{zz}}$, and $t_{\mathrm{rz}}$ are given by:

(12) $$\begin{array}{cc} t_{\mathrm{rr}}= & \mu \left[{\left(\frac{\partial u_{1}}{\partial R}\right)}^{2}+{\left(\frac{\partial u_{1}}{\partial Z}\right)}^{2}\right]-2C_{2}\left[4{\left(\frac{\partial u_{1}}{\partial R}\right)}^{2}+{\left(\frac{\partial w_{1}}{\partial R}+\frac{\partial u_{1}}{\partial Z}\right)}^{2}\right],\\ t_{\theta \theta }= & \left(\mu -8C_{2}\right){\left(\frac{u_{1}}{R}\right)}^{2},\\ t_{\mathrm{zz}}= & \mu \left[{\left(\frac{\partial w_{1}}{\partial Z}\right)}^{2}+{\left(\frac{\partial w_{1}}{\partial R}\right)}^{2}\right]-2C_{2}\left[4{\left(\frac{\partial w_{1}}{\partial Z}\right)}^{2}+{\left(\frac{\partial w_{1}}{\partial R}+\frac{\partial u_{1}}{\partial Z}\right)}^{2}\right],\\ t_{\mathrm{rz}}= & \mu \left[\frac{\partial u_{1}}{\partial R}\frac{\partial w_{1}}{\partial R}+\frac{\partial u_{1}}{\partial Z}\frac{\partial w_{1}}{\partial Z}\right]-4C_{2}\left[\frac{\partial u_{1}}{\partial R}+\frac{\partial w_{1}}{\partial Z}\right]\left[\frac{\partial w_{1}}{\partial R}+\frac{\partial u_{1}}{\partial Z}\right]. \end{array}$$

For axial symmetry, the equations of equilibrium for the symmetric contravariant stress T referred to the deformed coordinate system can be written as:

(13) $$\begin{array}{c} \frac{\partial T_{\mathrm{rr}}}{\partial r}+\frac{\partial T_{\mathrm{rz}}}{\partial z}+\frac{T_{\mathrm{rr}}-T_{\theta \theta }}{r}=0,\\ \frac{\partial T_{\mathrm{rz}}}{\partial r}+\frac{\partial T_{\mathrm{zz}}}{\partial z}+\frac{T_{\mathrm{rz}}}{r}=0. \end{array}$$

Using the expansions in powers of ε and noting the derivatives:

(14) $$\begin{array}{c} \frac{\partial }{\partial r}=\left(1-\varepsilon \frac{\partial u_{1}}{\partial R}\right)\frac{\partial }{\partial R}-\varepsilon \frac{\partial w_{1}}{\partial R}\frac{\partial }{\partial Z},\\ \frac{\partial }{\partial z}=\left(1-\varepsilon \frac{\partial w_{1}}{\partial Z}\right)\frac{\partial }{\partial Z}-\varepsilon \frac{\partial u_{1}}{\partial Z}\frac{\partial }{\partial R}, \end{array}$$

the first-order equations of equilibrium take the forms:

(15) $$\begin{array}{c} \frac{\partial T_{\mathrm{rr}}^{\left(1\right)}}{\partial R}+\frac{\partial T_{\mathrm{rz}}^{\left(1\right)}}{\partial Z}+\frac{T_{\mathrm{rr}}^{\left(1\right)}-T_{\theta \theta }^{\left(1\right)}}{R}=0,\\ \frac{\partial T_{\mathrm{rz}}^{\left(1\right)}}{\partial R}+\frac{\partial T_{\mathrm{zz}}^{\left(1\right)}}{\partial Z}+\frac{2T_{\mathrm{rz}}^{\left(1\right)}}{R}=0, \end{array}$$

and the second-order equations of equilibrium take the forms:

(16) $$\begin{array}{c} \frac{\partial T_{\mathrm{rr}}^{\left(2\right)}}{\partial R}+\frac{\partial T_{\mathrm{rz}}^{\left(2\right)}}{\partial Z}+\frac{T_{\mathrm{rr}}^{\left(2\right)}-T_{\theta \theta }^{\left(2\right)}}{R}=H_{R}\left(R,Z\right),\\ \frac{\partial T_{\mathrm{rz}}^{\left(2\right)}}{\partial R}+\frac{\partial T_{\mathrm{zz}}^{\left(2\right)}}{\partial Z}+\frac{T_{\mathrm{rz}}^{\left(2\right)}}{R}=H_{Z}\left(R,Z\right), \end{array}$$

where:

(17) $$\begin{array}{c} H_{R}\left(R,Z\right)=\frac{\partial u_{1}}{\partial R}\frac{\partial T_{\mathrm{rr}}^{\left(1\right)}}{\partial R}+\frac{\partial w_{1}}{\partial R}\frac{\partial T_{\mathrm{rr}}^{\left(1\right)}}{\partial Z}+\frac{\partial w_{1}}{\partial Z}\frac{\partial T_{\mathrm{zz}}^{\left(1\right)}}{\partial Z}+\frac{\partial u_{1}}{\partial Z}\frac{\partial T_{\mathrm{rz}}^{\left(1\right)}}{\partial R}+\frac{u_{1}}{R^{2}}\left(T_{\mathrm{rr}}^{\left(1\right)}-T_{\theta \theta }^{\left(1\right)}\right),\\ H_{Z}\left(R,Z\right)=\frac{\partial u_{1}}{\partial R}\frac{\partial T_{\mathrm{rz}}^{\left(1\right)}}{\partial R}+\frac{\partial w_{1}}{\partial R}\frac{\partial T_{\mathrm{rz}}^{\left(1\right)}}{\partial Z}+\frac{\partial w_{1}}{\partial Z}\frac{\partial T_{\mathrm{zz}}^{\left(1\right)}}{\partial Z}+\frac{\partial u_{1}}{\partial Z}\frac{\partial T_{\mathrm{zz}}^{\left(1\right)}}{\partial R}+\frac{u_{1}}{R^{2}}T_{\mathrm{rz}}^{\left(1\right)}. \end{array}$$

The second-order equations of equilibrium can be expressed in the forms:

(18) $$\begin{array}{c} -\frac{\partial p_{2}}{\partial R}+\mu \left(2\frac{\partial^{2}u_{2}}{\partial R^{2}}+\frac{\partial^{2}w_{2}}{\partial R\partial Z}+\frac{\partial^{2}u_{2}}{\partial Z^{2}}+\frac{2}{R}\frac{\partial u_{2}}{\partial R}-\frac{2u_{2}}{R^{2}}\right)+\frac{\partial t_{\mathrm{rr}}}{\partial R}+\frac{\partial t_{\mathrm{rz}}}{\partial Z}+\frac{t_{\mathrm{rr}}-t_{\theta \theta }}{R}=H_{R}\left(R,Z\right),\\ -\frac{\partial p_{2}}{\partial Z}+\mu \left(2\frac{\partial^{2}w_{2}}{\partial Z^{2}}+\frac{\partial^{2}u_{2}}{\partial R\partial Z}+\frac{\partial^{2}w_{2}}{\partial R^{2}}+\frac{1}{R}\frac{\partial u_{2}}{\partial Z}+\frac{1}{R}\frac{\partial w_{2}}{\partial R}\right)+\frac{\partial t_{\mathrm{rz}}}{\partial R}+\frac{\partial t_{\mathrm{zz}}}{\partial Z}+\frac{t_{\mathrm{rz}}}{R}=H_{Z}\left(R,Z\right). \end{array}$$

## 3. Spencer’s displacement functions

In the formulation of the *first-order* problem in incompressible elasticity, Spencer introduced the approach involving Stokes’ stream function, which satisfies the incompressibility condition for any choice of the function. This approach for the first-order problem bears a similarity with the problem of slow viscous flow for Newtonian fluids in terms of Stokes’ stream function (Lamb [115], Happel and Brenner [116], Langlois and Deville [117], Constantinescu [118]). This aspect has been observed by many researchers including Lord Rayleigh [119], Goodier [120], Hill [121], Prager [122], Adkins [123], Collins [124], and Richards [125]. The extension of the approach to the formulation of problems in second-order elasticity for incompressible elastic materials is *not straightforward* and techniques have to be developed to formulate the expressions for the second-order displacement field in terms of the *second-order displacement function* in such way that the second-order incompressibility condition can be satisfied.

Considering the first-order problem, we introduce a displacement function $\Psi_{1}(R,Z)$ such that the first-order displacement components $u_{1}(R,Z)$ and $w_{1}(R,Z)$ are given by:

(19) $$u_{1}=\frac{1}{R}\frac{\partial \Psi_{1}}{\partial Z};\;w_{1}=-\frac{1}{R}\frac{\partial \Psi_{1}}{\partial R}.$$

Considering these representations and first-order stresses (9) and the first-order equations of equilibrium, we arrive at the following partial differential equations governing the first-order displacement function $\Psi_{1}(R,Z)$ and the first-order isotropic stress $p_{1}(R,Z)$, i.e.,

(20) $$E^{4}\Psi_{1}\left(R,Z\right)=0;\;\nabla^{2}p\left(R,Z\right)=0,$$

where $E^{2}$ and $\nabla^{2}$ are, respectively, Stokes’ operator and Laplace’s operator defined by:

(21) $$\begin{array}{cc} E^{2}= & \frac{\partial^{2}}{\partial R^{2}}-\frac{1}{R}\frac{\partial }{\partial R}+\frac{\partial^{2}}{\partial Z^{2}};\;E^{4}=E^{2}E^{2},\\ \nabla^{2}= & \frac{\partial^{2}}{\partial R^{2}}+\frac{1}{R}\frac{\partial }{\partial R}+\frac{\partial^{2}}{\partial Z^{2}}. \end{array}$$

The extension of the displacement approach to the formulation of the second-order problem requires a representation that can exactly satisfy the second-order incompressibility condition given by equation (6). Methods that involve other techniques in terms of displacement and stress functions were proposed by Green and Spratt [80] and Chan and Carlson [83], but Spencer’s formulation retains the basic displacement function approach adopted for the first-order problem; the representation of the second-order displacement components in terms of the second-order displacement function have the forms:

(22) $$u_{2}=\frac{1}{R}\frac{\partial \Psi_{2}}{\partial Z}+\frac{u_{1}}{2}\left(\frac{\partial u_{1}}{\partial R}-\frac{\partial w_{1}}{\partial Z}\right);\;w_{2}=-\frac{1}{R}\frac{\partial \Psi_{1}}{\partial R}+u_{1}\frac{\partial w_{1}}{\partial R}.$$

As was indicated by Selvadurai and Spencer [85], there are other representations of the type (22) that can satisfy the second-order incompressibility condition (6) and they are admissible without exception. The second-order equations of equilibrium (18) can now be reduced to forms in terms of the second-order displacement function $\Psi_{2}(R,Z)$ and the second-order isotopic stress $p_{2}(R,Z)$ in the forms:

(23) $$\begin{array}{c} -\frac{\partial p_{2}}{\partial R}+\mu \left(\frac{1}{R}\frac{\partial }{\partial Z}\left(E^{2}\Psi_{2}\right)\right)=F_{R}\left(R,Z\right),\\ -\frac{\partial p_{2}}{\partial Z}-\mu \left(\frac{1}{R}\frac{\partial }{\partial R}\left(E^{2}\Psi_{2}\right)\right)=F_{Z}\left(R,Z\right), \end{array}$$

where:

(24) $$\begin{array}{cc} F_{R}\left(R,Z\right)= & H_{R}\left(R,Z\right)-\left(\frac{\partial {t\prime }_{\mathrm{rr}}}{\partial R}+\frac{\partial {t\prime }_{\mathrm{rz}}}{\partial Z}+\frac{{t\prime }_{\mathrm{rr}}-{t\prime }_{\theta \theta }}{R}\right),\\ F_{Z}\left(R,Z\right)= & H_{Z}\left(R,Z\right)-\left(\frac{\partial {t\prime }_{\mathrm{rz}}}{\partial R}+\frac{\partial {t\prime }_{\mathrm{zz}}}{\partial Z}+\frac{{t\prime }_{\mathrm{rz}}}{R}\right), \end{array}$$

and

(25) $$\begin{array}{c} {t\prime }_{\mathrm{rr}}=\mu \left\{u_{1}\left(\frac{u_{1}}{R^{2}}-2\frac{\partial^{2}w_{1}}{\partial R\partial Z}\right)+3{\left(\frac{\partial u_{1}}{\partial R}\right)}^{2}+{\left(\frac{\partial u_{1}}{\partial Z}\right)}^{2}\right\}-2C_{2}\left\{4{\left(\frac{\partial u_{1}}{\partial R}\right)}^{2}+{\left(\frac{\partial u_{1}}{\partial Z}+\frac{\partial w_{1}}{\partial R}\right)}^{2}\right\},\\ {t\prime }_{\theta \theta }=-2\mu \left(\frac{u_{1}}{R}\right)\left(\frac{\partial w_{1}}{\partial Z}\right)-8C_{2}{\left(\frac{u_{1}}{R}\right)}^{2},\\ {t\prime }_{\mathrm{zz}}=\mu \left\{2u_{1}\frac{\partial^{2}w_{1}}{\partial R\partial Z}+2\left(\frac{\partial u_{1}}{\partial Z}\right)\left(\frac{\partial w_{1}}{\partial R}\right)+{\left(\frac{\partial w_{1}}{\partial R}\right)}^{2}+{\left(\frac{\partial w_{1}}{\partial Z}\right)}^{2}\right\}-2C_{2}\left\{4{\left(\frac{\partial w_{1}}{\partial Z}\right)}^{2}+{\left(\frac{\partial u_{1}}{\partial Z}+\frac{\partial w_{1}}{\partial R}\right)}^{2}\right\},\\ {t\prime }_{\mathrm{rz}}=\mu \left\{u_{1}\left(\frac{\partial^{2}u_{1}}{\partial R\partial Z}+\frac{\partial^{2}w_{1}}{\partial R^{2}}\right)+2\left(\frac{\partial u_{1}}{\partial R}\right)\left(\frac{\partial w_{1}}{\partial R}\right)\right\}+4C_{2}\left\{\frac{u_{1}}{R}\left(\frac{\partial u_{1}}{\partial Z}+\frac{\partial w_{1}}{\partial R}\right)\right\}. \end{array}$$

By successively eliminating $p_{2}(R,Z)$ and $\Psi_{2}(R,Z)$ from equation (23), we obtain the following partial differential equations:

(26) $$\begin{array}{cc} E^{4}\Psi_{2}\left(R,Z\right)= & \frac{R}{\mu }\left(\frac{\partial F_{R}}{\partial Z}-\frac{\partial F_{Z}}{\partial R}\right),\\ \nabla^{2}p_{2}\left(R,Z\right)= & -\left(\frac{\partial F_{R}}{\partial R}+\frac{F_{R}}{R}+\frac{\partial F_{Z}}{\partial Z}\right), \end{array}$$

which govern the axisymmetric second-order problem for an incompressible elastic material. For the solution of specific boundary value problems, both traction and/or displacement boundary conditions need to be specified on the deformed boundaries.

### 3.1. Boundary conditions

The development of solutions to the first-order problem is straightforward and either traction or displacement boundary conditions could be specified on surfaces referred to the undeformed configuration. In the case of the second-order problem, the displacement and traction boundary conditions have to be specified in relation to surfaces that are prescribed on the deformed body. The components of the traction vector on a surface in the deformed body, resolved in the r- and z-directions are:

(27) $$F_{r}=n_{r}T_{\mathrm{rr}}+n_{z}T_{\mathrm{rz}};\;F_{z}=n_{r}T_{\mathrm{rz}}+n_{z}T_{\mathrm{zz}},$$

where:

(28) $$n_{r}^{2}+n_{z}^{2}=1;\;\frac{n_{r}}{n_{z}}=\frac{\frac{\partial F}{\partial r}}{\frac{\partial F}{\partial z}},$$

and the traction boundary conditions are normally specified in relation to a surface in the deformed body defined by:

(29) $$F\left(r,z\right)=\tilde{F}\left(R,Z\right)=0.$$

From equation (28), the normal to the deformed surface has components, $n_{r}^{\left(1\right)}+\varepsilon n_{r}^{\left(2\right)}$ and $n_{z}^{\left(1\right)}+\varepsilon n_{z}^{\left(2\right)}$ to order ε, where:

(30) $$\begin{array}{c} {\left(n_{r}^{\left(1\right)}+\varepsilon n_{r}^{\left(2\right)}\right)}^{2}+{\left(n_{z}^{\left(1\right)}+\varepsilon n_{z}^{\left(2\right)}\right)}^{2}=1,\\ \frac{n_{r}^{\left(1\right)}+\varepsilon n_{r}^{\left(2\right)}}{n_{z}^{\left(1\right)}+\varepsilon n_{z}^{\left(2\right)}}=\frac{\frac{\partial \tilde{F}}{\partial R}-\varepsilon \left(\frac{\partial u_{1}}{\partial R}\frac{\partial \tilde{F}}{\partial R}+\frac{\partial w_{1}}{\partial R}\frac{\partial \tilde{F}}{\partial Z}\right)}{\frac{\partial \tilde{F}}{\partial Z}-\varepsilon \left(\frac{\partial u_{1}}{\partial Z}\frac{\partial \tilde{F}}{\partial R}+\frac{\partial w_{1}}{\partial Z}\frac{\partial \tilde{F}}{\partial Z}\right)}. \end{array}$$

Using these relationships, the expressions (27) can be expressed as:

(31) $$\begin{array}{c} F_{r}=\varepsilon \left\{n_{r}^{\left(1\right)}T_{\mathrm{rr}}^{\left(1\right)}+n_{z}^{\left(1\right)}T_{\mathrm{rz}}^{\left(1\right)}\right\}+\varepsilon^{2}\left\{n_{r}^{\left(1\right)}T_{\mathrm{rr}}^{\left(2\right)}+n_{z}^{\left(1\right)}T_{\mathrm{rz}}^{\left(2\right)}+n_{r}^{\left(2\right)}T_{\mathrm{rr}}^{\left(1\right)}+n_{z}^{\left(2\right)}T_{\mathrm{rz}}^{\left(1\right)}\right\},\\ F_{z}=\varepsilon \left\{n_{r}^{\left(1\right)}T_{\mathrm{rz}}^{\left(1\right)}+n_{z}^{\left(1\right)}T_{\mathrm{zz}}^{\left(1\right)}\right\}+\varepsilon^{2}\left\{n_{r}^{\left(1\right)}T_{\mathrm{rr}}^{\left(2\right)}+n_{z}^{\left(1\right)}T_{\mathrm{zz}}^{\left(2\right)}+n_{r}^{\left(2\right)}T_{\mathrm{rz}}^{\left(1\right)}+n_{z}^{\left(2\right)}T_{\mathrm{zz}}^{\left(1\right)}\right\}. \end{array}$$

## 4. Kelvin’s problem

**Figure 1.**
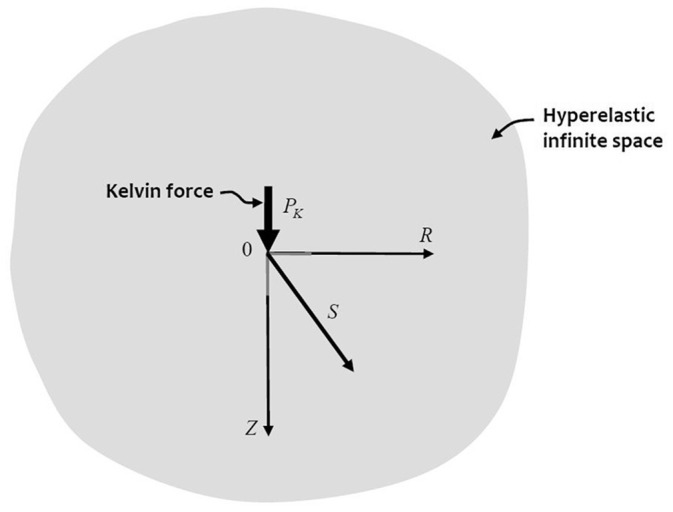
Kelvin’s problem.

The state of stress in an elastic solid of infinite extent subjected to a concentrated force of magnitude $P_{K}$ applied at the origin of coordinates of a system of cylindrical polar coordinates was first solved by Lord Kelvin [97] (Figure 1). When the line of action of the concentrated force coincides with the Z-axis of the cylindrical polar coordinate system (R,Θ,Z), the problem is axisymmetric and the solution to the first-order or classical elasticity problem can be found in a variety of ways, which are summarized in texts on classical elasticity and its applications [98–105]. For example, if Love’s strain potential approach is applied to the solution of Kelvin’s problem with axial symmetry, the analysis requires the use of the strain potential Ω(R,Z), which satisfies the bi-harmonic equation:

(32) $$\nabla^{4}\Omega \left(R,Z\right)=0,$$

where the displacement and stress components are given by:

(33) $$\begin{array}{c} 2\mu u_{1}=-\frac{\partial^{2}\Omega }{\partial R\partial Z}\;;\;2\mu w_{1}=2\left(1-\nu \right)\nabla^{2}\Omega -\frac{\partial^{2}\Omega }{\partial Z^{2}},\\ T_{\mathrm{rr}}^{\left(1\right)}=\frac{\partial }{\partial Z}\left(\nu \;\nabla^{2}\Omega -\frac{\partial^{2}\Omega }{\partial R^{2}}\right)\;;\;T_{\theta \theta }^{\left(1\right)}=\frac{\partial }{\partial Z}\left(\nu \;\nabla^{2}\Omega -\frac{1}{R}\frac{\partial \Omega }{\partial R}\right)\;;\;T_{\mathrm{zz}}^{\left(1\right)}=\frac{\partial }{\partial Z}\left((2-\nu )\;\nabla^{2}\Omega -\frac{\partial^{2}\Omega }{\partial Z^{2}}\right),\\ T_{\mathrm{rz}}^{\left(1\right)}=\frac{\partial }{\partial R}\left(\left(1-\nu \right)\;\nabla^{2}\Omega -\frac{\partial^{2}\Omega }{\partial Z^{2}}\right), \end{array}$$

and ν is Poisson’s ratio.

A function that furnishes a solution to Kelvin’s problem is Love’s potential:

(34) $$\Omega \left(R,Z\right)=B{\left(R^{2}+Z^{2}\right)}^{1/2},$$

where B is an arbitrary constant. Restricting the analysis to the case of an incompressible elastic material, we can show that the first-order displacement and stress components take the forms:

(35) $$\begin{array}{cc} 2\mu u_{1}= & B\frac{\mathrm{RZ}}{{\left(R^{2}+Z^{2}\right)}^{3/2}};\;2\mu w_{1}=B\frac{R^{2}+2Z^{2}}{{\left(R^{2}+Z^{2}\right)}^{3/2}},\\ T_{\mathrm{rr}}^{\left(1\right)}= & B\left(-3\frac{R^{2}Z}{{\left(R^{2}+Z^{2}\right)}^{5/2}}\right);\;T_{\theta \theta }^{\left(1\right)}=0,\\ T_{\mathrm{zz}}^{\left(1\right)}= & B\left(-3\frac{Z^{3}}{{\left(R^{2}+Z^{2}\right)}^{5/2}}\right);\;T_{\mathrm{rz}}^{\left(1\right)}=B\left(-3\frac{RZ^{2}}{{\left(R^{2}+Z^{2}\right)}^{5/2}}\right). \end{array}$$

The constant B can be determined by calculating the resultant force acting on a cylindrical or spherical surface enclosing the origin. This gives:

(36) $$B=\frac{P_{K}}{4\pi }.$$

It is noted that the first-order displacement and stress fields reduce to zero as (R,Z)→∞, and they are singular as (R,Z)→0. This is a constraint of the first-order solution for Kelvin’s problem. The only means of maintaining the first-order solution bounded as (R,Z)→0 is to physically exclude the origin of coordinates through the provision of either a cavity or an inclusion of finite dimension. This approach was used by Chadwick and Trowbridge [126], Selvadurai [90,127,128] and Selvadurai and Dasgupta [129] by including either a rigid spherical or spheroidal inclusion at the origin and satisfying the appropriate displacement and/or traction boundary conditions on the surface of the inclusion. A second-order elasticity problem of the centrally loaded spherical rigid inclusion analogue of Kelvin’s problem was examined by Selvadurai [90]. In order to develop the second-order solution to the localized Kelvin force problem, we utilize the first-order solution given by equation (35). Avoiding details of lengthy algebraic manipulations, it can be shown that the partial differential equation governing the second-order displacement function takes the form:

(37) $$E^{4}\Psi_{2}=-\frac{3P_{K}^{2}}{4\pi^{2}\mu^{2}}\left(\frac{2C_{2}}{\mu }-1\right)\frac{ZR^{2}}{{\left(R^{2}+Z^{2}\right)}^{4}}.$$

The particular integral of equation (37) can be obtained by converting the equation into a spherical polar coordinate form, where:

(38) $$\begin{array}{c} R=S\sin\Theta ;\;Z=S\cos\Theta ;\;S={\left(R^{2}+Z^{2}\right)}^{1/2}\\ E^{2}=\left(\frac{\partial^{2}}{\partial S^{2}}+\frac{1}{S^{2}}\frac{\partial^{2}}{\partial \Theta^{2}}-\frac{\cot\Theta }{S^{2}}\frac{\partial }{\partial \Theta }\right). \end{array}$$

It can be shown that the relevant expressions for the displacement and stress components derived from the particular solution of equation (37) are:

(39) $$\begin{array}{cc} u_{2p}= & \frac{K^{2}}{{\left(R^{2}+Z^{2}\right)}^{3}}\left\{\left(\frac{2C_{2}}{\mu }\right)\left(2R^{3}-6RZ^{2}\right)+\frac{1}{2}\left(-R^{3}+3RZ^{2}\right)\right\},\\ w_{2p}= & \frac{K^{2}}{{\left(R^{2}+Z^{2}\right)}^{3}}\left\{\left(\frac{2C_{2}}{\mu }\right)\left(-4Z^{3}+4R^{2}Z\right)-2R^{2}Z\right\},\\ T_{\mathrm{rr}}^{(2)p}= & K^{2}\mu \left\{\left(\frac{2C_{2}}{\mu }\right)\left(\frac{-14R^{4}+68R^{2}Z^{2}-2Z^{4}}{{\left(R^{2}+Z^{2}\right)}^{4}}\right)+\left(\frac{3R^{4}-18R^{2}Z^{2}+3Z^{4}}{{\left(R^{2}+Z^{2}\right)}^{4}}\right)+\left(\frac{R^{6}+Z^{6}}{{\left(R^{2}+Z^{2}\right)}^{5}}\right)\right\},\\ T_{\theta \theta }^{(2)p}= & K^{2}\mu \left\{\left(\frac{2C_{2}}{\mu }\right)\left(\frac{2R^{2}-2Z^{2}}{{\left(R^{2}+Z^{2}\right)}^{3}}\right)+\left(\frac{-R^{2}+4Z^{2}}{{\left(R^{2}+Z^{2}\right)}^{3}}\right)\right\},\\ T_{\mathrm{zz}}^{(2)p}= & K^{2}\mu \left\{\left(\frac{2C_{2}}{\mu }\right)\left(\frac{6R^{4}-56R^{2}Z^{2}+22Z^{4}}{{\left(R^{2}+Z^{2}\right)}^{4}}\right)+\left(\frac{-4R^{4}+20R^{2}Z^{2}}{{\left(R^{2}+Z^{2}\right)}^{4}}\right)+\left(\frac{R^{6}+9R^{4}Z^{2}+12R^{2}Z^{4}+4Z^{6}}{{\left(R^{2}+Z^{2}\right)}^{5}}\right)\right\},\\ T_{\mathrm{rz}}^{(2)p}= & K^{2}\mu \left\{\left(\frac{2C_{2}}{\mu }\right)\left(\frac{44Z^{3}R-40ZR^{3}}{\left(R^{2}+Z^{2}\right){)}^{4}}\right)+\left(\frac{17ZR^{3}-10Z^{3}R}{{\left(R^{2}+Z^{2}\right)}^{4}}\right)\right\}. \end{array}$$

where:

(40) $$K=\frac{P_{K}}{8\pi \mu }.$$

The displacement and stress components (39) satisfy the first- and second-order incompressibility conditions in equation (6) and the second-order equations of equilibrium (2), where:

(41) $$\begin{array}{cc} G\left(R,Z\right)= & K^{2}\left(\frac{-R^{2}+3Z^{2}}{{\left(R^{2}+Z^{2}\right)}^{3}}\right),\\ H_{R}\left(R,Z\right)= & K^{2}\mu \left(\frac{-42Z^{2}R+6R^{3}}{{\left(R^{2}+Z^{2}\right)}^{4}}\right);\;H_{Z}\left(R,Z\right)=K^{2}\mu \left(\frac{-36Z^{3}+12ZR^{2}}{{\left(R^{2}+Z^{2}\right)}^{4}}\right), \end{array}$$

and

(42) $$-p_{2}\left(R,Z\right)=K^{2}\mu \left\{\left(\frac{2C_{2}}{\mu }\right)\left(\frac{14Z^{2}-2R^{2}}{{\left(R^{2}+Z^{2}\right)}^{3}}\right)\right\}.$$

We note that the second-order solution is symmetric about Z=0, with $w_{2p}(R,0)=0$ and $T_{\mathrm{rz}}^{(2)p}(R,0)=0$. Therefore, on any closed surface enclosing the origin, the resultant traction in the *Z*-direction is zero, and there is no second-order contribution to $P_{K}$.

It may be noted that the order of any singularities that may exist in the first-order solution [130–132] is usually increased in the second-order solution (England [133]). The second-order plane strain problem of an infinite plane containing an elliptic cavity was examined by Lianis [134] using the complex variable-based methodology presented by Adkins et al. [71]. The study was extended by De Hoff [135] to include the problem of an infinite plane containing an elliptical hole and subjected to an oblique uniaxial far field tension. The article by Varley and Cumberbatch [136] examines the elliptical cavity problem for a Fritz John-type hyperelastic material from which the stress magnification for a crack can be recovered. Both analyses indicate that in the limit of the elliptical cavity degenerating to a crack, the order of the singularity is increased for the second-order solution. Similar conclusions are presented by Knesl and Semela [137] for the case of the second-order results for a Westergaard-type crack. The finite deformation of a crack into the shape of a wedge is also treated by Blatz [138] and the stress states corresponding to the infinitesimal and finite strain solutions are compared. A related problem of inhomogeneous deformations of hyperelastic wedges is discussed by Rajagopal and Carroll [139]. A useful discussion of the limits of successive approximate techniques similar to that encountered in the Poincare–Lighthill–Kuo method in aerofoil theory was presented by Tsien [140]. The mathematical modelling of cracks in hyperelastic materials was examined in a series of elegant articles by Knowles and Sternberg [141–145] and Knowles [146]. Complimentary investigations of the plane crack problem were also discussed by Amazigo [147], Lo [148], Le and Stumpf [149], Geubelle and Knauss [150] and Tarantino [151]. Finite deformation analyses at the crack tip located at a bimaterial elastic interface consisting of hyperelastic materials were also developed by Knowles and Sternberg [152]. Ravichandran and Knauss [153], Herrmann [154, 155], Geubelle and Knauss [156, 157], Gao and Shi [158] and Gao [159] to investigate the oscillatory nature of the singularity at the tip of a crack located in a bimaterial region under far-field stresses. The vanishing of the oscillatory stress singularity can result from either the incompressibility constraint usually associated with hyperelastic materials and/or the finite curvature that the crack tip can acquire from the finite deformation.

### 4.1. Solutions of the homogeneous equation

Considering the homogeneous equation (37), expressed in spherical polar coordinates, we can write:

(43) $$E^{4}\Psi_{2h}=\left(\frac{\partial^{2}}{\partial S^{2}}+\frac{1}{S^{2}}\frac{\partial^{2}}{\partial \Theta^{2}}-\frac{\cot\Theta }{S^{2}}\frac{\partial }{\partial \Theta }\right)\left(\frac{\partial^{2}}{\partial S^{2}}+\frac{1}{S^{2}}\frac{\partial^{2}}{\partial \Theta^{2}}-\frac{\cot\Theta }{S^{2}}\frac{\partial }{\partial \Theta }\right)\Psi_{2h}=0.$$

Considering the substitution ρ=cosΘ , equation (40) can be written as:

(44) $$\left(\frac{\partial^{2}}{\partial S^{2}}+\frac{\left(1-\rho^{2}\right)}{S^{2}}\frac{\partial^{2}}{\partial \rho^{2}}\right)\left(\frac{\partial^{2}\Psi_{2p}}{\partial S^{2}}+\frac{\left(1-\rho^{2}\right)}{S^{2}}\frac{\partial^{2}\Psi_{2p}}{\partial \rho^{2}}\right)=0.$$

We seek solutions of equation (44) of the form:

(45) $$\Psi_{2p}=\frac{f\left(\rho \right)}{S},$$

which can be used to reduce equation (44) to:

(46) $$\left(\left(1-\rho^{2}\right)\frac{d^{2}}{d\rho^{2}}+12\right)\left(\left(1-\rho^{2}\right)\frac{d^{2}}{d\rho^{2}}+2\right)f\left(\rho \right)=0.$$

We note that the operators in equation (46) commute, i.e.,

(47) $$\left(\left(1-\rho^{2}\right)\frac{d^{2}}{d\rho^{2}}+2\right)\left(\left(1-\rho^{2}\right)\frac{d^{2}}{d\rho^{2}}+12\right)f\left(\rho \right)=0.$$

Solving the resulting ordinary differential equations (ODEs), we obtain the following homogeneous solutions:

(48) $$\begin{array}{c} \Psi_{2h}=A\left(\frac{R^{2}}{{\left(R^{2}+Z^{2}\right)}^{3/2}}\right)+B\left(\frac{2Z}{\left(R^{2}+Z^{2}\right)}+\frac{R^{2}}{{\left(R^{2}+Z^{2}\right)}^{3/2}}\log_{e}\left(\frac{\sqrt{R^{2}+Z^{2}}+Z}{\sqrt{R^{2}+Z^{2}}-Z}\right)\right)+C\left(\frac{R^{2}\left(R^{2}-4Z^{2}\right)}{{\left(R^{2}+Z^{2}\right)}^{5/2}}\right)\\ +D\left(\frac{26ZR^{2}-4Z^{3}}{{\left(R^{2}+Z^{2}\right)}^{2}}+\frac{3R^{2}\left(R^{2}-4Z^{2}\right)}{{\left(R^{2}+Z^{2}\right)}^{5/2}}\log_{e}\left(\frac{\sqrt{R^{2}+Z^{2}}+Z}{\sqrt{R^{2}+Z^{2}}-Z}\right)\right), \end{array}$$

where A,B,C, and D are the arbitrary constants. Evaluating the displacement and stress components from equation (48) we note that, in order to maintain the second-order displacement component $u_{2h}$ and the second-order shear stress component $T_{\mathrm{rz}}^{(2)h}$ to be bounded as R→0, we require B=D=0. The remaining solutions give the following second-order displacement and stress components:

(49) $$\begin{array}{c} u_{2h}=A\left(-\frac{3\mathrm{RZ}}{{\left(R^{2}+Z^{2}\right)}^{5/2}}\right)+C\left(\frac{2R^{3}Z-3RZ^{3}}{{\left(R^{2}+Z^{2}\right)}^{7/2}}\right),\\ w_{2h}=A\left(\frac{R^{2}-2Z^{2}}{{\left(R^{2}+Z^{2}\right)}^{5/2}}\right)+C\left(\frac{-2Z^{4}+3Z^{2}R^{2}}{{\left(R^{2}+Z^{2}\right)}^{7/2}}\right), \end{array}$$

and

(50) $$\begin{array}{c} T_{\mathrm{rr}}^{(2)h}=A\left(\frac{24R^{2}Z-6Z^{3}}{{\left(R^{2}+Z^{2}\right)}^{7/2}}\right)+C\left(\frac{-22R^{4}Z+46Z^{3}R^{2}-2Z^{5}}{{\left(R^{2}+Z^{2}\right)}^{9/2}}\right),\\ T_{\theta \theta }^{(2)h}=A\left(\frac{6R^{2}Z-6Z^{3}}{{\left(R^{2}+Z^{2}\right)}^{7/2}}\right)+C\left(\frac{-2R^{4}Z-4Z^{3}R^{2}-2Z^{5}}{{\left(R^{2}+Z^{2}\right)}^{9/2}}\right),\\ T_{\mathrm{zz}}^{(2)h}=A\left(\frac{-18R^{2}Z+12Z^{3}}{{\left(R^{2}+Z^{2}\right)}^{7/2}}\right)+C\left(\frac{6R^{4}Z-48Z^{3}R^{2}+16Z^{5}}{{\left(R^{2}+Z^{2}\right)}^{9/2}}\right),\\ T_{\mathrm{rz}}^{(2)h}=A\left(\frac{-6R^{3}+24Z^{2}R}{{\left(R^{2}+Z^{2}\right)}^{7/2}}\right)+C\left(\frac{2R^{5}-36Z^{2}R^{3}+32Z^{4}R}{{\left(R^{2}+Z^{2}\right)}^{9/2}}\right). \end{array}$$

**Figure 2.**
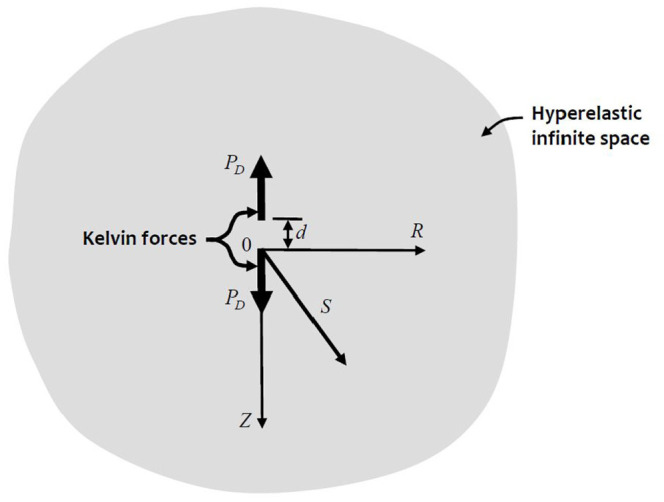
The doublet problem.

We observe that since $u_{2h}(R,0)=0$ and $T_{\mathrm{zz}}^{(2)h}(R,0)=0$, the homogeneous solution will involve only states of deformation and stress that are *asymmetric* about Z=0, which can be caused only by resultant forces similar in character to the Kelvin force and this includes higher-order singularities in both the displacement and stress fields. The first-order displacement function for a concentrated force acting at the interior of an elastic infinite space of infinite extent is given by:

(51) $${\left(\Psi_{1}\right)}_{\mathrm{Kelvin}\;\mathrm{force}}=C_{1}^{*}\left(\frac{R^{2}}{{\left(R^{2}+Z^{2}\right)}^{1/2}}\right),$$

where $C_{1}^{*}$ is a constant. The first-order displacement potential for a doublet (see section 5), which consist of two collinear Kelvin forces separated by a small distance d (Figure 2), is given by the partial derivative of equation (51) with respect to Z, i.e.,

(52) $${\left(\Psi_{1}\right)}_{\mathrm{Doublet}}=C_{2}^{*}\left(\frac{R^{2}Z}{{\left(R^{2}+Z^{2}\right)}^{3/2}}\right),$$

where $C_{2}^{*}$ is a constant. The first-order displacement potential for a combination of doublets (i.e., a doublet in *tension* in close proximity to a doublet in *compression*) can be obtained by taking the partial derivative of equation (52) with respect to Z, i.e.,

(53) $${\left(\Psi_{1}\right)}_{\mathrm{Doublet}}=C_{3}^{*}\left(\frac{R^{2}}{{\left(R^{2}+Z^{2}\right)}^{3/2}}-\frac{3R^{2}Z^{2}}{{\left(R^{2}+Z^{2}\right)}^{5/2}}\right),$$

where $C_{3}^{*}$ is a constant. The result (53) has a form similar to a homogeneous solution that can be obtained by suitably adjusting the coefficients A and C in the reduced solution for equation (48). Therefore, the second-order solutions derived from the homogeneous solution (45) become relevant only if additional second-order local forces are applied in the vicinity of the Kelvin force. It can be concluded that the second-order displacement and stress fields corresponding to the Kelvin force problem are obtained from the particular solution of equation (37). These solutions agree with the results obtained by Carroll and Rooney [94] for the second-order Kelvin’s problem, using a Love Strain potential approach along with Signorini’s compatibility condition.

## 5. The doublet problem

Kelvin’s classical elasticity solution for the single point force acting at the interior of an elastic solid of infinite extent can be successfully used to find novel solutions of the equations of classical elasticity corresponding to other singularities. One such problem, described by Love [96] as a “double force without moment,” is obtained by superposing on the Kelvin force problem (the force denoted by $P_{D}$) a point force of equal magnitude, acting in the opposite direction but located at a small distance d from the origin, in the negative *Z*-direction (Figure 2). Since the second force is acting in the opposite direction and considering d to be infinitesimally small, the first-order solutions to the doublet problem is obtained by replacing any term in the solution to Kelvin’s problem, e.g., f(R,Z) by f+(∂f/∂Z)d (see, e.g., [99]).

For an incompressible elastic material, the first-order displacement and stress components for the doublet problem can be written as:

(54) $$\begin{array}{cc} u_{1}= & \tilde{C}\frac{\left(-R^{3}+2RZ^{2}\right)}{{\left(R^{2}+Z^{2}\right)}^{5/2}};\;w_{1}=\tilde{C}\frac{\left(2Z^{3}-ZR^{2}\right)}{{\left(R^{2}+Z^{2}\right)}^{5/2}},\\ T_{\mathrm{rr}}^{\left(1\right)}= & 2\tilde{C}\mu \left(\frac{3R^{4}-12R^{2}Z^{2}}{{\left(R^{2}+Z^{2}\right)}^{7/2}}\right);\;T_{\theta \theta }^{\left(1\right)}=0,\\ T_{\mathrm{zz}}^{\left(1\right)}= & 2\tilde{C}\mu \left(\frac{9R^{2}Z^{2}-6Z^{4}}{{\left(R^{2}+Z^{2}\right)}^{7/2}}\right);\;T_{\mathrm{rz}}^{\left(1\right)}=2\tilde{C}\mu \left(\frac{6R^{3}Z-9RZ^{3}}{{\left(R^{2}+Z^{2}\right)}^{5/2}}\right), \end{array}$$

where:

(55) $$\tilde{C}=\frac{P_{D}d}{8\pi \mu }.$$

Omitting details, the partial differential equation governing the second-order displacement function $\Psi_{2}(R,Z)$ takes the form:

(56) $$E^{4}\Psi_{2}=\frac{{\tilde{C}}^{2}}{{\left(R^{2}+Z^{2}\right)}^{6}}\left[144\left(\frac{2C_{2}}{\mu }\right)\left(R^{4}Z-4R^{2}Z^{3}\right)-27\left(3R^{4}Z-2R^{2}Z^{2}+5Z^{5}\right)\right].$$

The second-order expressions for the displacement and stress components obtained from the particular solution of equation (56) can be written in the forms:

(57) $$\begin{array}{cc} u_{2p}= & \frac{{\tilde{C}}^{2}}{{\left(R^{2}+Z^{2}\right)}^{5}}\left[\left(\frac{2C_{2}}{\mu }\right)\left(-5R^{5}+47R^{3}Z^{2}-20RZ^{4}\right)-\left(\frac{3}{8}\left(-4R^{5}+52R^{3}Z^{2}-16RZ^{4}\right)\right)\right]\\ w_{2p}= & \frac{{\tilde{C}}^{2}}{{\left(R^{2}+Z^{2}\right)}^{5}}\left[\left(\frac{2C_{2}}{\mu }\right)\left(-20R^{4}Z+44R^{2}Z^{3}-8Z^{5}\right)-\left(\frac{3}{8}\left(-24R^{4}Z+48R^{2}Z^{3}\right)\right)\right]\\ T_{\mathrm{rr}}^{(2)p}= & \frac{{\tilde{C}}^{2}}{{\left(R^{2}+Z^{2}\right)}^{6}}\left[2C_{2}\left(48R^{6}-684R^{4}Z^{2}+528R^{2}Z^{4}\right)+\mu \left(-12R^{6}+324R^{4}Z^{2}-237R^{2}Z^{4}+12Z^{6}\right)\right]\\ T_{\theta \theta }^{(2)p}= & \frac{{\tilde{C}}^{2}}{{\left(R^{2}+Z^{2}\right)}^{6}}\left[2C_{2}\left(72R^{4}Z^{2}+72R^{2}Z^{4}\right)+\mu \left(3R^{6}-36R^{4}Z^{2}-27R^{2}Z^{4}+12Z^{6}\right)\right]\\ T_{\mathrm{zz}}^{(2)p}= & \frac{{\tilde{C}}^{2}}{{\left(R^{2}+Z^{2}\right)}^{6}}\left[2C_{2}\left(-30R^{6}+540R^{4}Z^{2}-618R^{2}Z^{4}+72Z^{6}\right)+\mu \left(18R^{6}-279R^{4}Z^{2}+300R^{2}Z^{4}+12Z^{6}\right)\right]\\ T_{\mathrm{rz}}^{(2)p}= & \frac{{\tilde{C}}^{2}}{{\left(R^{2}+Z^{2}\right)}^{6}}\left[2C_{2}\left(240R^{5}Z-80R^{3}Z^{3}+216RZ^{5}\right)+\mu \left(-111R^{5}Z+402R^{3}Z^{3}-72RZ^{5}\right)\right]. \end{array}$$

The second-order displacement and stress components (57) satisfy the second-order incompressibility condition (6) and the second-order equations of equilibrium (23), where:

(58) $$\begin{array}{cc} G\left(R,Z\right)= & {\tilde{C}}^{2}\left(\frac{3R^{4}-12R^{2}Z^{2}+12Z^{4}}{{\left(R^{2}+Z^{2}\right)}^{5}}\right),\\ H_{R}\left(R,Z\right)= & {\tilde{C}}^{2}\left(\frac{-54R^{5}+162R^{3}Z^{2}-324RZ^{4}}{{\left(R^{2}+Z^{2}\right)}^{6}}\right),\\ H_{Z}\left(R,Z\right)= & {\tilde{C}}^{2}\left(\frac{-108R^{4}Z+216R^{2}Z^{3}-216Z^{5}}{{\left(R^{2}+Z^{2}\right)}^{4}}\right). \end{array}$$

## 6. Boussinesq’s problem

**Figure 3.**
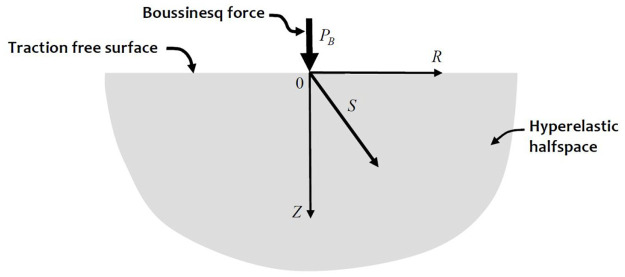
Boussinesq’s problem.

The solution to the problem of an isotropic elastic half-space that is subjected to a concentrated normal force at the origin of coordinates is identified as Boussinesq’s problem (Boussinesq [160]) (Figure 3). The solution to the linear elasticity problem can be obtained in diverse ways, including through the application of integral transform techniques [98–105, 161–163] and through the successive applications of superposition techniques involving Kelvin’s solution and Love’s strain potential technique [96]. The recent study [113] develops a solution to Boussinesq’s problem using Kelvin’s solution and dimensional considerations in choosing additional solutions required to render the surface of the half-space region traction free. While methods have been developed to apply Boussinesq’s solution to examine the decay in the distribution of stress in earth masses subjected to surface loads, the modifications proposed fail to satisfy the requirement of classical elasticity, particularly in terms of satisfying the Beltrami–Michell compatibility conditions [114].

The application of the theory of second-order elasticity to Boussinesq’s problem is a natural extension of the study dealing with Kelvin’s problem. The first-order solution for Boussinesq’s problem is identical in form to Kelvin’s solution given by equation (35) and the arbitrary constant corresponding to equation (36) is given by:

(59) $$B^{*}=\frac{P_{B}}{4\pi }.$$

The stress field given in equation (39) must satisfy the traction-free boundary conditions applicable to the deformed surface of the half-space region. The equation of the deformed surface of the half-space valid to order ε is given by:

(60) $$F\left(r,z\right)=z-\varepsilon w_{1}\left(R,Z\right)=\mathrm{const}.$$

The components of the unit normal in the *r*- and *z*-directions are given by:

(61) $$n=\frac{\left(-\varepsilon \frac{\partial w_{1}}{\partial r},0,\left\{1-\varepsilon \frac{\partial w_{1}}{\partial z}\right\}\right)}{\sqrt{\varepsilon^{2}{\left(\frac{\partial w_{1}}{\partial r}\right)}^{2}+\varepsilon^{2}{\left(1-\varepsilon \frac{\partial w_{1}}{\partial z}\right)}^{2}}},$$

which reduces to:

(62) $$n=\left(-\varepsilon \frac{\partial w_{1}}{\partial R},0,1\right).$$

The traction-free boundary conditions for the second-order stress components on the deformed surface of the half-space reduce to:

(63) $$\begin{array}{c} T_{\mathrm{rz}}^{\left(2\right)}-\frac{\partial w_{1}}{\partial R}T_{\mathrm{rr}}^{\left(1\right)}=0\;\mathrm{on}\;Z=0,\\ T_{\mathrm{zz}}^{\left(2\right)}-\frac{\partial w_{1}}{\partial R}T_{\mathrm{rz}}^{\left(1\right)}=0\;\mathrm{on}\;Z=0. \end{array}$$

Since $T_{\mathrm{rr}}^{\left(1\right)}=T_{\mathrm{rz}}^{\left(1\right)}=0\;\mathrm{on}\;Z=0$, the second-order stress field should satisfy the boundary conditions $T_{\mathrm{rz}}^{\left(2\right)}=T_{\mathrm{zz}}^{\left(2\right)}=0\;\mathrm{on}\;Z=0$. The results from the particular solution (39) give:

(64) $$\begin{array}{c} T_{\mathrm{rz}}^{\left(2\right)}=0\;\mathrm{on}\;Z=0,\\ T_{\mathrm{zz}}^{\left(2\right)}=\frac{3K^{2}}{R^{4}}\left(4C_{2}-\mu \right)\;\mathrm{on}\;Z=0. \end{array}$$

The first boundary condition of equation (64) is identically satisfied by the expression for $T_{\mathrm{rz}}^{(2)p}$ given by equation (39). The second boundary condition needs to be satisfied by selecting a suitable solution of the homogeneous equation of equation (37) that will have the correct distribution of $T_{\mathrm{zz}}^{(2)h}(R,0)$. The reduced form of equation (48) will not provide the necessary solution. The alternative is to use Love’s stress function approach and obtain the stress field that can satisfy the second boundary conditions of equation (64). Selvadurai [86] showed that a solution based on the Hankel integral transforms (Lamb [161], Sneddon [162,163], Terezawa [164]) does not provide an approach to satisfy the required traction-free boundary condition. A direct integration of Boussinesq’s solution applicable to a half-space is a possible approach. For example, the expression for the required homogeneous solution for the displacement $u_{2h}(R,Z)$ will give results of the type

(65) $$u_{2h}\left(R,Z\right)=\int_{0}^{2\pi }\int_{0}^{\infty }\frac{\mathrm{CZ}}{8\pi \mu {\left(R\prime \right)}^{4}}\frac{{\left\{R^{2}+{\left(R\prime \right)}^{2}-2\mathrm{RR}\cos\theta \prime \right\}}^{1/2}}{{\left\{Z^{2}+R^{2}+{\left(R\prime \right)}^{2}-2\mathrm{RR}\prime \cos\theta \prime \right\}}^{3/2}}R\prime \mathrm{dR}\prime d\theta \prime .$$

While this approach is feasible, the divergent integrals can only be evaluated numerically, which is a limitation. Also, consideration of a hemi-spherical inclusion at the surface of the half-space results in the first-order solution that is a series in the Legendre polynomials, which cannot be manipulated easily in constructing the second-order formulation. Subsequently, the second-order solution to Boussinesq’s problem for a concentrated normal force was provided in a compact and closed form by Carroll and Rooney [95], using the strain potential approach of Love [96].

## 7. Conclusion

The theory of second-order elasticity is a mathematically consistent theory for modeling hyperelastic materials undergoing moderately large strains, as opposed to either media exhibiting large deflections and rotations but with small strains or small deformations superposed on large. The expansion of the dependent variables in terms of a small non-dimensional parameter forms the basis of the approach and if a particular problem is formulated in a consistent manner, the small parameter will naturally evolve in the formulation. The solution of problems in second-order elasticity theory also becomes meaningful in terms of technological applications involving rubber-like elastic materials used as load bearing components or mountings. Also, when the incompressibility constraint is introduced, the strain energy function of the Mooney–Rivlin form can completely accommodate the second-order moderately large strain phenomena. The formulation and solution of problems in second-order elasticity can be facilitated through the use of complex potentials and complex variable theory, stress functions based on the adaptation of the functions proposed by Love, Neuber–Papkovich, and integral transform techniques. Spencer’s displacement function approach is also a convenient method for the treatment of the second-order incompressible elastic problem and reduces the governing partial differential equation for the function to a canonical linear form. A similar result is obtained for the second-order component of the isotropic stress. The displacement function approach is applied to develop solutions to certain axisymmetric localized loading problems of an infinite space and a half-space. The first-order problem corresponds to the classical elasticity solution and the order of the stress singularities present in the first-order problems are generally increased in the second-order problem. The issue of singularities in the first-order solution can be alleviated by selecting suitable boundary conditions where the loads are applied over finite regions. The ensuing formulations can involve algebraic complexity. In such cases the solution of the second-order problem entails a great deal of routine mathematical operations and computer-aided symbolic mathematical manipulation techniques can be used to solve the second-order problem with speed and accuracy.

## Appendix 1

The origins of AJM Spencer’s development of the displacement function approach for problems in second-order elasticity theory. The pages are excerpts taken from the hand-written notes of Tony Spencer, which formed the basis for the studies [85] and [86] and the former was written during his visit to the *Center for the Application of Mathematics* at Lehigh University in 1968.
